# Comparative In Vitro Antifungal Activity of Essential Oils, Plant Extracts, and Commercial Biological Products Against *Fusarium avenaceum* and *Alternaria alternata*

**DOI:** 10.3390/plants15172566

**Published:** 2026-08-24

**Authors:** Vytautas Bunevičius, Armina Morkeliūnė, Justina Griauzdaitė, Ingrida Mažeikienė, Alma Valiuškaitė, Neringa Rasiukevičiūtė

**Affiliations:** 1 Laboratory of Plant Protection, Institute of Horticulture, Lithuanian Research Centre for Agriculture and Forestry, Kaunas District, LT-54333 Babtai, Lithuania; vytautas.bunevicius@lammc.lt (V.B.); armina.morkeliune@lammc.lt (A.M.); justina.griauzdaite@lammc.lt (J.G.); alma.valiuskaite@lammc.lt (A.V.); 2 Department of Orchard Plant Genetics and Biotechnology, Institute of Horticulture, Lithuanian Research Centre for Agriculture and Forestry, Kaunas District, LT-54333 Babtai, Lithuania; ingrida.mazeikiene@lammc.lt

**Keywords:** clove, inhibition, plant essential oils, plant extracts, rosemary, thyme

## Abstract

Fungal pathogens, such as *Fusarium* spp. and *Alternaria* spp., cause substantial yield losses worldwide through root and fruit rot, wilting, and leaf and fruit spots, and increasing restrictions on chemical pesticides have intensified interest in sustainable alternatives such as essential oils, plant extracts, and microbial biocontrol agents. However, these three treatment categories are rarely compared directly under identical experimental conditions, which limits conclusions about their relative efficacy. This study directly compared, for the first time under the same in vitro conditions, the antifungal activity of peppermint and thyme essential oils, clove and rosemary plant extracts, and three commercial biological products (Mycostop, Asir Fruit, Aegis) against *Fusarium avenaceum* and *Alternaria alternata* using the agar incorporation method, with fungicidal versus fungistatic activity determined by reinoculation. Most treatments inhibited mycelial growth of both pathogens relative to the untreated control, although a few treatments (Asir Fruit at early DAI and lowest-concentration thyme EO) showed no inhibition or even slight stimulation of *A. alternata* growth. At 7 days after inoculation, thyme essential oil (1000 µL/L) and clove plant extract at all concentrations tested completely inhibited both pathogens, and reinoculation confirmed that clove extract alone was fungicidal, likely reflecting its high eugenol content and consequent irreversible membrane damage; all other treatments were fungistatic, consistent with reversible effects on membrane permeability. Peppermint essential oil (2000 µL/L) inhibited *F. avenaceum* and *A. alternata* by 96% and 73%, respectively, while rosemary extract was comparatively weaker (57% and 21%). Among the commercial products, Mycostop, which contains *Streptomyces griseoviridis*, was the most effective, plausibly reflecting sustained antagonistic activity beyond metabolite secretion alone. These findings indicate that clove extract and thyme oil match or exceed commercial biocontrol products in vitro and warrant evaluation under greenhouse and field conditions as candidates for integrated disease management.

## 1. Introduction

Modern agriculture faces many challenges each year due to pathogenic fungi, bacteria, viruses, parasitic plants and nematodes. Diseases caused by these organisms lead to significant yield losses, which can range from 20% to 40% annually [1]. Thousands of fungal species have been identified as plant pathogens and are considered the dominant causal agents of plant diseases worldwide [2]. Pesticides are commonly used to manage pathogenic fungal problems. However, given their adverse effects and increasing restrictions on their use, this encourages research on alternative control strategies. Currently, promising alternatives to chemical pesticides include essential oils, plant extracts, bacterial and fungal species such as *Bacillus* and *Streptomyces* isolates, and others [3,4,5,6,7].

*Fusarium avenaceum* and *Alternaria alternata* are widespread in occurrence, are of economic importance worldwide, and are also relevant as plant pathogens in Lithuania. *Fusarium* spp. is a widespread genus of fungi, widely distributed in soil [8]. It causes significant yield losses in horticulture and agriculture, and the mycotoxins it produces have harmful effects on humans and animals [8,9]. Members of the genus *Fusarium* cause root and fruit rot, wilting, leaf and fruit spots, and apple dieback and contribute to apple replant disease [10,11]. *Fusarium* species cause significant economic damage across diverse plants, with *F. graminearum* and *F. oxysporum* recognized as two of the world’s top 10 fungal plant pathogens [12]. *F. avenaceum* is a wound-infecting pathogen and has been identified as the primary species responsible for most cases of fruit rot in apples in Croatia [13]. In recent years, head blight caused by *F. graminearum* has become one of the main cereal diseases in Lithuania [14].

Over 95% of *Alternaria* species are classified as plant pathogens, which can cause infections in a wide range of plants, including potatoes, tomatoes, cruciferous crops, blueberries, pomegranates, and apples [15]. Their effects can cause spots, blight, and rot in different parts of the plant [16]. Apple *Alternaria* blotch, caused by the *Alternaria alternata*, is considered one of the most destructive fungal diseases of apple worldwide [17]. Numerous *Alternaria* species have been identified as pathogens affecting various vegetables in Lithuania, including cabbage, tomatoes, onions, carrots and cucumbers. These include species such as *A. cucumerina*, *A. brassicae*, *A. solani*, *A. dauci*, and *A. alternata* [18,19]. *Alternaria* fruit spot is also a common post-harvest problem for apples in Lithuania [20].

Plant essential oils (EOs) and extracts (PEs) are increasingly recognized as promising alternatives to chemical pesticides due to their safety, environmental friendliness, and biodegradability. Biological pesticides obtained from plants contain alkaloids, lignans, glycosides, terpenes, terpenoids, phenols and other metabolites, which are friendly alternatives to chemical pesticides. The substances in EO and PE contain individual biological components with highly active compounds. The terpenes and terpenoids they contain can damage fungal cell walls, leading to cell death or inhibiting sporulation and germination in a wide range of fungal species [3,21]. The antifungal efficacy of peppermint essential oil is attributed to its terpenes, which interact with the lipid fraction of the plasma membrane, leading to membrane leakage. Meanwhile, terpenoids inhibit oxygen uptake and oxidative phosphorylation [22]. The inhibitory efficacy of essential oils, such as pine, geranium, spruce, patchouli, coriander, eucalyptus, fennel, hops, thymus, lavender, thyme and peppermint, has been demonstrated in various in vitro studies against various pathogenic fungi [3,18,23,24]. *Mentha × piperita* L. EO has been reported to exhibit inhibitory effects against *Rhizoctonia solani*, *Penicillium expansum*, and *Alternaria alternata* [25]. Rosemary and clove EOs and PEs show inhibitory effects against various genera of fungi: *Fusarium*, *Aspergillus*, *Botrytis*, *Colletotrichum* and *Mucor* [4]. Notably, the resistance of fungal isolates may vary due to their development under different agroclimatic conditions, and the composition of EO and PE may also differ depending on growth conditions, harvesting stage, and extraction method [22]. Therefore, it is essential to conduct research using extracts obtained under different conditions and to investigate fungi cultivated under diverse agroclimatic conditions.

Various microbiological products containing beneficial microorganisms play an important role in crop protection and stress resilience. Nevertheless, the efficacy of individual microbial treatments in mitigating biotic and abiotic stresses is highly variable, being governed not only by environmental conditions but also by the specific traits and adaptability of the selected microorganism strains. This double dependency largely explains the differential performance frequently observed among distinct biological formulations [26]. The genus *Bacillus* is widely known as plant-growth-promoting bacteria due to its ability to fix nitrogen, convert insoluble phosphorus and potassium into plant-available forms, and produce phytohormones [5]. In addition, *Bacillus* species are well known for inhibiting pathogenic microorganisms, mainly through the production of siderophores, antibiotics, and lytic enzymes [27,28,29]. They can inhibit pathogens both directly, via antimicrobial compounds, and indirectly, by inducing plant resistance and competing for resources [30]. Despite these properties, not all *Bacillus* isolates exhibit inhibitory activity against certain pathogens. Studies have reported that, of 65 isolates investigated, only 16 inhibited *Fusarium* spp. Therefore, it is important to find suitable isolates for the suppression of relevant pathogens [31]. Effective inhibition of mycelial growth has been observed using different strains of *B. velezensis* and *B. halotolerans* against *Botrytis cinerea*, *Fusarium culmorum*, *Monilinia fructicola*, *Monilinia frutigena*, and *Monilinia laxa* [32].

Arbuscular mycorrhizal fungi (AMF) are among the components of commercial products. AMF are commonly used in this case to reduce plant damage through multiple mechanisms. They limit resources available to plant pathogens by occupying space, enhancing plant defense responses, and stimulating the activity of beneficial microorganisms. AMF comprises a wide variety of fungi, including genera such as *Funneliformis*, *Rhizoglomus*, *Sclerocarpum*, *Septoglomus*, and many others [6,33]. Another microorganism already used in biological products is *Streptomyces* spp. The antibiotics, hydrolytic enzymes, and other metabolites they produce inhibit the growth of various pathogenic microorganisms by damaging cell walls and membranes, interfering with intracellular processes, and disrupting cell division. *S. griseoviridis*, *S. hydrogenans*, and *S. corchorusii* have been reported to inhibit pathogens of different fungal genera [34]. Another widely recognized group of microorganisms that compete with phytopathogens is *Trichoderma* spp. Species of this genus compete with pathogens for nutrients and space within plant tissues, produce cell wall-degrading enzymes and inhibitory secondary metabolites, and can parasitize pathogens by forming appressoria, thereby exploiting pathogen resources and suppressing their growth [35,36]. The commercial biological product Aegis contains *Rhizoglomus irregularis*, and Asir Fruit contains *Funneliformis mosseae*, a plant-growth-promoting organism that indirectly inhibits the activity of surrounding pathogens [6,37,38]. *Trichoderma koningii*, present in Asir Fruit, inhibits pathogens both directly and indirectly by producing antimicrobial compounds and enzymes [39,40]. The use of diverse antagonistic isolates, the development of combined microbial products, and the selection of strains adapted to suppress different pathogens remain important directions for future research. Future studies should evaluate the effectiveness of these products against diverse pathogen isolates from different geographical regions under greenhouse and field conditions.

This study aimed to evaluate, using the agar incorporation method, the in vitro antifungal activity of peppermint and thyme essential oils, clove and rosemary plant extracts, and three commercial products (Mycostop, Asir Fruit, Aegis) against *Fusarium avenaceum* and *Alternaria alternata* and to determine whether each treatment was fungicidal or fungistatic. Previous studies have typically evaluated essential oils, plant extracts, and commercial products separately, often under different experimental conditions and against different pathogens, making direct comparisons difficult. By testing all three categories against the same isolates under identical in vitro conditions, this study provides a direct comparison of their antifungal activity, based on mycelial growth inhibition and the outcome of reinoculation, and offers a foundation for developing alternative, EO-based and PE-based pesticides.

## 2. Results

### 2.1. In Vitro Inhibitory Effect of Different Treatments

Statistical comparison of the inhibitory effects of the tested commercial treatments and the highest concentrations of EO and PE at 7 DAI is presented in Table 1. Among the plant extracts, clove plant extract (CPE) exhibited the highest antifungal activity (100% against both isolates), whereas rosemary plant extract (RPE) was the least effective (57.34% and 21.26% against *F. avenaceum* and *A. alternata*, respectively). Among the essential oils, thyme essential oil (TEO) showed the strongest inhibitory effect, achieving complete inhibition at the highest concentration tested. Peppermint essential oil (PEO) exhibited 95.63% and 72.95% inhibition against *F. avenaceum* and *A. alternata*, respectively. Among the commercial products, Mycostop was the most effective (inhibition reached 83.46% and 94.68% against *F. avenaceum* and *A. alternata*, respectively), although its antifungal activity against *F. avenaceum* remained lower than that of CPE and TEO. Aegis and Asir Fruit showed comparatively weaker antifungal effects than the most effective plant-derived treatments.

The inhibitory effects of the different treatments on mycelial growth of the two fungal isolates were evaluated, and the results are presented in Table 2. Seven days after inoculation (DAI), complete inhibition of *F. avenaceum* was achieved with TEO at 1000 µL/L and with CPE at all tested concentrations. For *A. alternata*, complete inhibition at 7 DAI was likewise achieved with TEO at 1000 µL/L and with CPE at all tested concentrations. Among the commercial products, Mycostop exhibited the highest inhibitory activity (94.68%). The lowest inhibition was observed with TEO at 200 µL/L, resulting in growth exceeding the control and indicating fungal growth exceeding that of the untreated control. Further analytical details of the results and their graphical presentation are provided in the following sections.

### 2.2. Inhibitory Effect of Peppermint Essential Oil

**Figure 1:**
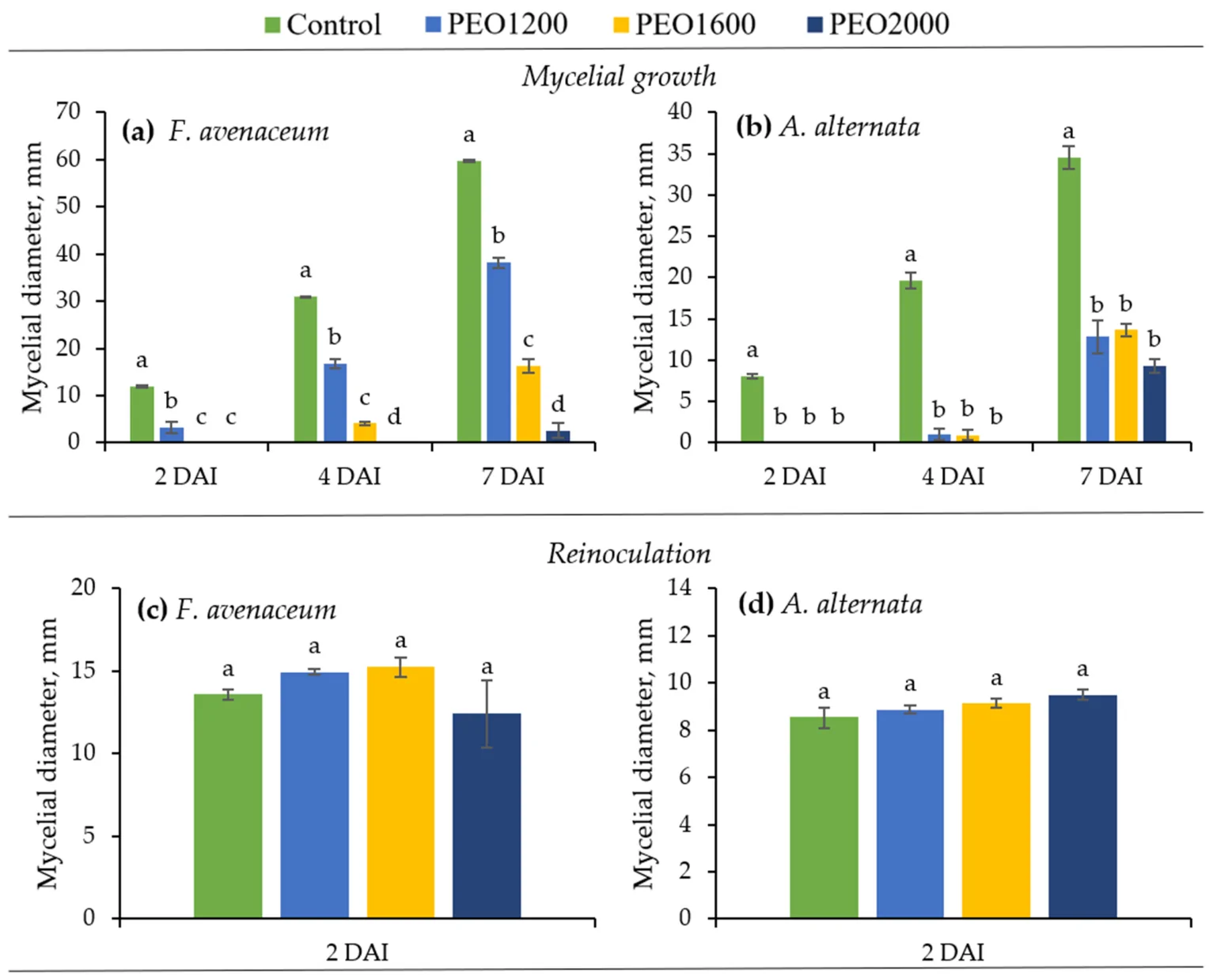
Mycelial growth of *F. avenaceum* (**a**) and *A. alternata* (**b**) treated with peppermint EO at different concentrations 2, 4, and 7 days after inoculation and the results of reinoculation (**c**,**d**) at 2 DAI. Data are presented as mean ± standard error. Significant differences (*p* < 0.05) were identified within the same evaluation day by Duncan’s post hoc test and are indicated by different letters.

The inhibitory effect of peppermint essential oil against *F. avenaceum* was evaluated, and reinoculation was assessed afterwards. The results are presented in Figure 1a,c. Inhibition at 2 DAI at a PEO concentration of 1200 μL/L reached 73%. At higher concentrations, complete inhibition of mycelial growth was observed. After 4 days of inoculation, the inhibition of *F. avenaceum* reached around 46% and 87% at PEO concentrations of 1200 μL/L and 1600 μL/L, respectively. Mycelial growth was not visible at the highest concentration. Inhibition at 7 DAI was 36%, 73% and 96%, respectively, at increasing concentrations. Following pathogen inhibition, a reinoculation was conducted, and the results showed that at 2 DAI, no significant differences in mycelial growth were observed across concentrations compared to the control (fungistatic effect).

The inhibitory effect of PEO and the reinoculation study were also performed against *A. alternata* (Figure 1b,d). Complete inhibition of mycelium growth was observed at all concentrations tested at 2 DAI. A minor mycelial growth was recorded at 4 DAI at the lowest two concentrations, with approximately 95% inhibition. At a PEO concentration of 2000 μL/L, the complete inhibition of mycelium growth was still visible. No significant differences were observed across the 1200–2000 µL/L range, and inhibition at 7 DAI was approximately 63%, 60%, and 73%, respectively. Reinoculation showed that *A. alternata* was not suppressed and was able to continue mycelial development after transfer of the fungal mycelium. At 2 DAI, no significant differences in mycelial growth were observed at the tested concentrations. These results indicate that the EO exhibited no fungicidal activity on the tested concentrations.

### 2.3. Inhibitory Effect of Thyme Essential Oil

**Figure 2:**
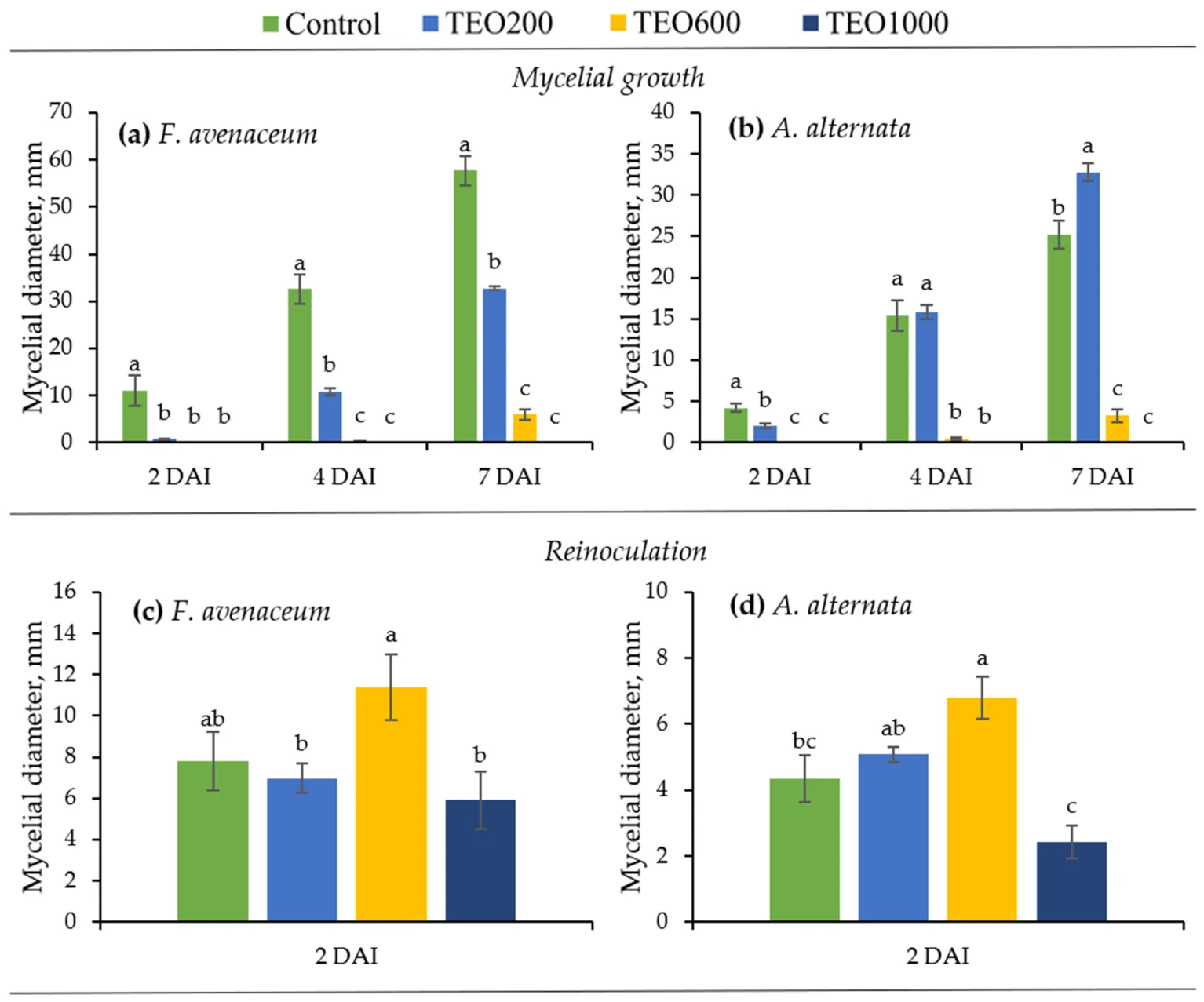
Mycelial growth of *F. avenaceum* (**a**) and *A. alternata* (**b**) treated with thyme EO at different concentrations 2, 4, and 7 days after inoculation and results of reinoculation (**c**,**d**) at 2 DAI. Data are presented as mean ± standard error. Significant differences (*p* < 0.05) were identified within the same evaluation day by Duncan’s post hoc test and are indicated by different letters.

Thyme essential oil (TEO) exhibited inhibitory effects against the tested isolates in vitro. *F. avenaceum* inhibition (Figure 2a,c) at a TEO concentration of 200 µL/L reached 94%, 67%, and 43% on each measurement day, respectively. At higher concentrations, more intense inhibition was observed: no mycelial growth was visible at 2 DAI. At 4 DAI, the inhibition at the two highest concentrations reached 99% and 100%, respectively. Meanwhile, at 7 DAI and TEO concentrations of 600 and 1000 µL/L, inhibition still reached 90% and 100%, respectively. After pathogen reinoculation, results at 2 DAI showed no significant inhibition in mycelial growth across all concentrations compared to the control, suggesting that the treatment is fungistatic.

The inhibitory effect of TEO was also observed against *A. alternata* (Figure 2b,d). At 2 DAI, inhibition at concentrations of 200, 600, and 1000 µL/L reached 53%, 100%, and 100%, respectively. At 4 DAI, the TEO 200 µL/L concentration showed no significant difference compared to the control, whereas at higher concentrations, inhibition remained at 97% and 100%, respectively. At 7 DAI, the TEO 200 µL/L concentration showed pronounced mycelial growth, which may be attributed to hormesis. On the final day of evaluation, TEO inhibition at 600 and 1000 µL/L reached 87% and 100%, respectively. Reinoculation results indicate that the TEO exhibited no fungicidal activity at the tested concentrations.

### 2.4. Inhibitory Effect of Clove Plant Extract

**Figure 3:**
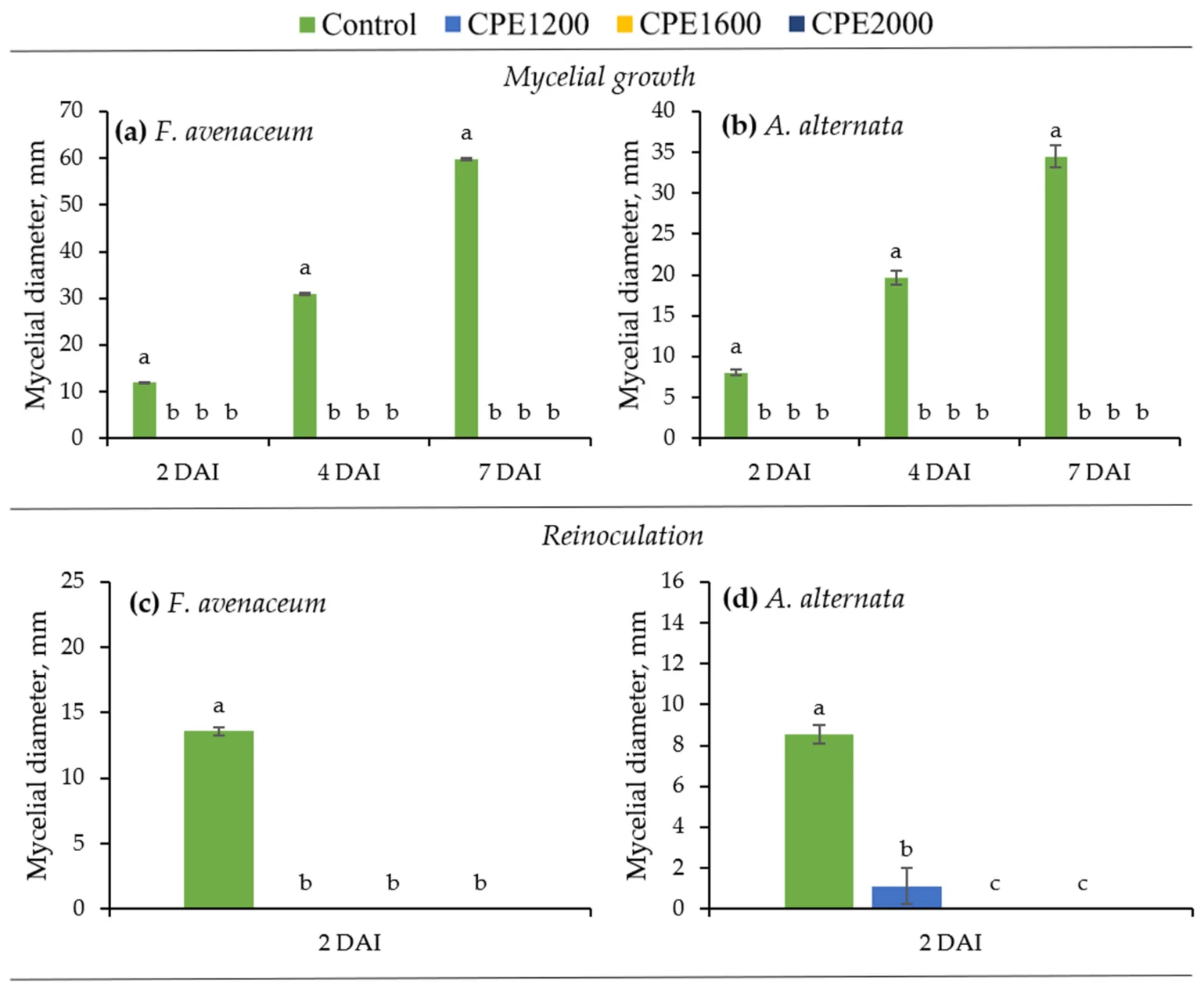
Mycelial growth of *F. avenaceum* (**a**) and *A. alternata* (**b**) treated with clove PE at different concentrations 2, 4, and 7 days after inoculation and results of reinoculation (**c**,**d**) at 2 DAI. Data are presented as mean ± standard error. Significant differences (*p* < 0.05) were identified within the same evaluation day by Duncan’s post hoc test and are indicated by different letters.

Inhibitory effects were observed not only with essential oils but also with plant extracts. Clove plant extract (CPE) completely inhibited *F. avenaceum* mycelial growth across all days after inoculation, regardless of concentration (Figure 3a,c). Complete inhibition of mycelial growth by CPE was also observed after reinoculation, indicating the fungicidal effect of the extract at the tested concentrations.

The CPE exhibited a similar effect against *A. alternata* (Figure 3b,d). Compared to the control, mycelial growth inhibition reached 100% at 2, 4, and 7 DAI at all concentrations tested. Differences were observed only after reinoculation: at a CPE concentration of 1200 µL/L, the extract showed a fungistatic effect, with inhibition reaching 87%. At higher concentrations, reinoculation results indicated that the CPE had a fungicidal effect.

### 2.5. Inhibitory Effect of Rosemary Plant Extract

**Figure 4:**
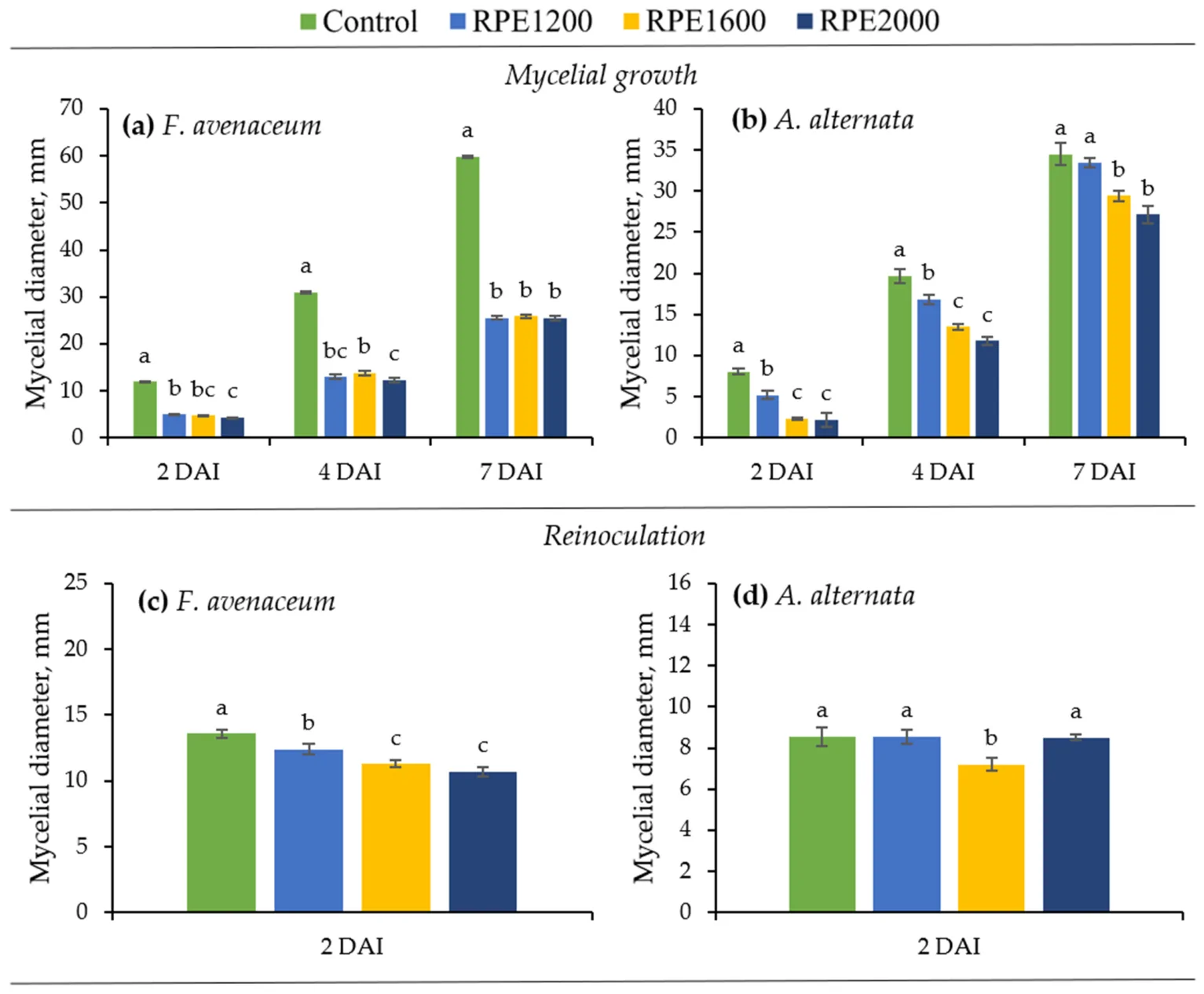
Mycelial growth of *F. avenaceum* (**a**) and *A. alternata* (**b**) treated with rosemary PE at different concentrations 2, 4, and 7 days after inoculation and results of reinoculation (**c**,**d**) at 2 DAI. Data are presented as mean ± standard error. Significant differences (*p* < 0.05) were identified within the same evaluation day by Duncan’s post hoc test and are indicated by different letters.

Rosemary plant extract (RPE) exhibited inhibitory effects against *F. avenaceum* (Figure 4a,c), with inhibition at 2 DAI increasing to around 59%, 60%, and 64%, respectively, as concentrations increased. RPE at 4 DAI, inhibition was 58%, 55%, and 60%, respectively. By 7 DAI, no significant differences were observed among the concentrations, and inhibition remained at approximately 57% across all concentrations. Reinoculation results indicate that RPE exhibited fungistatic activity at the tested concentrations.

The inhibitory effect of RPE against *A. alternata* was tested and the reinoculation study was also performed against *A. alternata* (Figure 4b,d). At a concentration of 1200 µL/L, inhibition of fungal growth reached 35% and 14% at 2 and 4 DAI, respectively, while by 7 DAI, no significant differences were observed compared to the control. At 1600 µL/L, RPE inhibition on the respective evaluation days was 70%, 31%, and 15%. No significant differences were observed between the 1600 µL/L and 2000 µL/L concentrations of RPE, with inhibition by 2000 µL/L remaining at similar levels across the evaluation days (72%, 40%, and 21%). The reinoculation results showed that RPE had no fungicidal activity at the concentrations tested.

### 2.6. Inhibitory Effect of Biological Products

**Figure 5:**
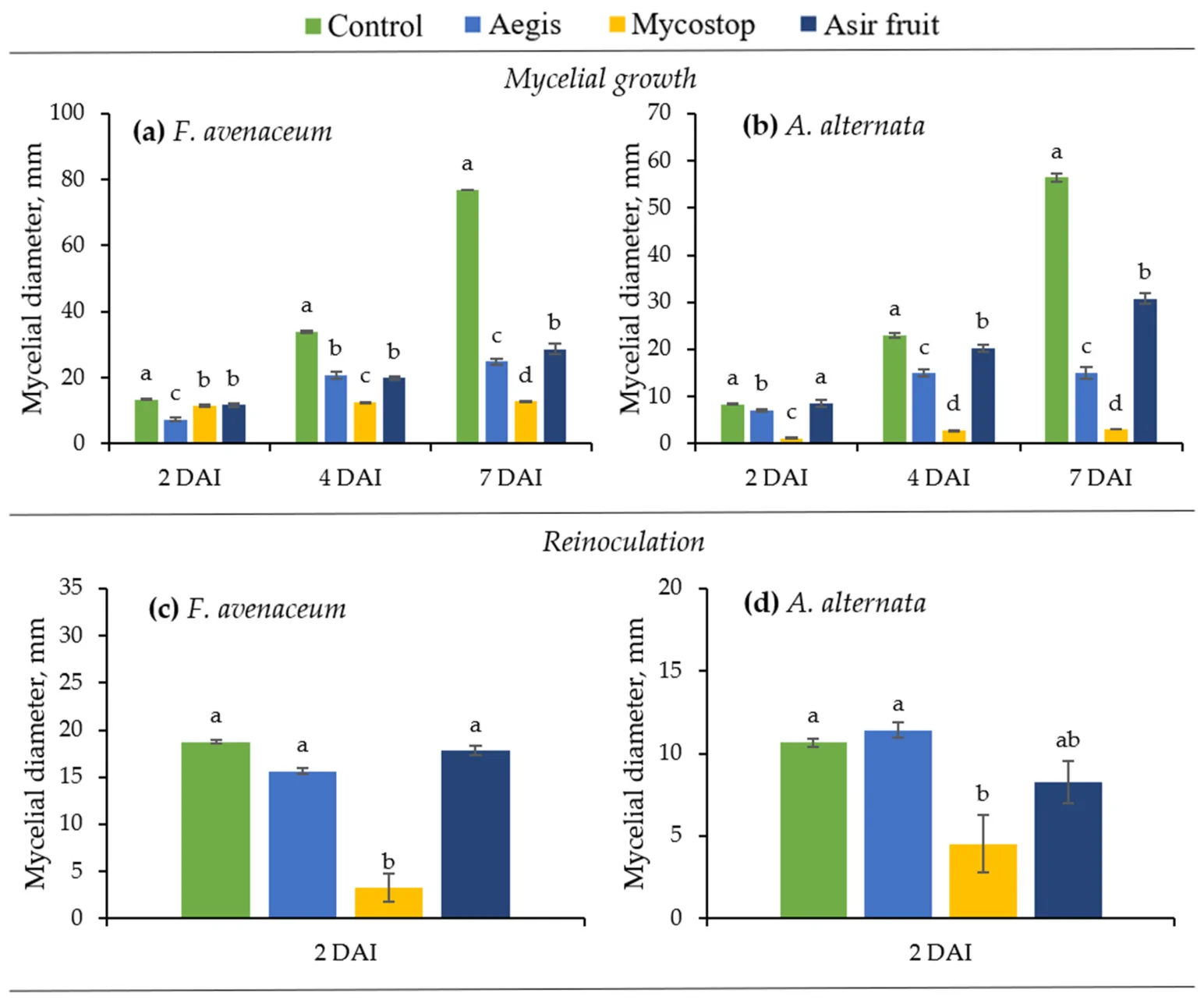
Mycelial growth of *F. avenaceum* (**a**) and *A. alternata* (**b**) treated with different commercial products 2, 4, and 7 days after inoculation and results of reinoculation (**c**,**d**) at 2 DAI. Data are presented as mean ± standard error. Significant differences (*p* < 0.05) were identified within the same evaluation day by Duncan’s post hoc test and are indicated by different letters.

After analyzing the results, it was observed that the three tested commercial biological products had inhibitory effects against *F. avenaceum*. The experimental results are presented in Figure 5a,c. At 2 DAI, the Mycostop and Asir Fruit products showed similar inhibitory effects, reaching 15% and 13%, respectively. More effective inhibition was observed with Aegis, reaching 45%. After 4 days of inoculation, Mycostop showed the most effective inhibition at 63%. The inhibition was 39% and 41% using Aegis and Asir Fruit, respectively. At 7 DAI, Mycostop remained the most effective, with inhibitory effects of 83%, 68%, and 63% for Mycostop, Aegis, and Asir Fruit, respectively. The reinoculation results showed that Aegis and Asir Fruit had no significant effect on mycelial growth inhibition at 2 DAI. In comparison, Mycostop exhibited increased antifungal activity, reaching 83% inhibition at 2 DAI. This demonstrates that Mycostop exerts a sustained fungistatic effect with carry-over antagonistic activity rather than a strictly transient fungistatic response.

Analysis of the results showed that all three tested commercial biological products exhibited inhibitory activity against *A. alternata* (Figure 5b,d). Mycostop was the most effective on all study days. Its inhibitory effects at 2, 4, and 7 DAI reached 86%, 88%, and 95%, respectively. The inhibitory effect of Aegis over the past days was 15%, 35% and 73%, respectively. At 2 DAI, Asir Fruit showed no significant differences from the control. However, inhibition was observed at 4 and 7 DAI, with 12% and 46%, respectively. After evaluating the reinoculation results, Mycostop showed inhibitory activity, reaching 58% at 2 DAI, compared to the control. Other treatments showed no significant differences relative to control, and all exhibited a fungistatic effect.

## 3. Discussion

Although chemical pesticides remain widely used in plant protection, their use is reducing under increasingly strict European Union regulation, while effective alternatives remain limited. This has increased interest in plant-based and microbial products as alternatives for plant protection. We therefore evaluated, for the first time under identical in vitro conditions, three functionally distinct categories of alternative treatment—essential oils (*Mentha × piperita* L. and *Thymus vulgaris* L.), plant extracts (*Rosmarinus officinalis* L. and *Syzygium aromaticum* L.), and commercial biological products—against *F. avenaceum* and *A. alternata*, two pathogens of major economic importance in Lithuania and worldwide [8]. Many treatments inhibited mycelial growth relative to the untreated control, but their underlying modes of action, and consequently their fungistatic or fungicidal character, differed substantially between categories. The compositional data for the thyme EO [41] oil and other oils in our study are consistent with previous reports, although in different proportions. Thyme (*Thymus vulgaris* L.) collected in Romania during the flowering stage was found to contain 47.59% thymol, 30.90% γ-terpinene, and 8.41% p-cymene [42]. The composition of *Thymus* and *Mentha* species essential oils and different extracts is strongly influenced by day length, temperature, and other environmental conditions. High-quality *M. × piperita* L. EO typically contains 44–55% menthol and 15–32% menthone. The EO of peppermint grown in Turkey (clone-3) was reported to contain 23.08% menthone and 37.02% menthol, differing from higher-menthol chemotypes reported in other regions [43]. In comparison, the essential oil used in our study was characterized by a high menthone content (44.56%) and a low menthol content (7.95%) [24].

This membrane-level mode of action is consistent with the broad antifungal spectrum reported for *Mentha × piperita* L. oil against species such as *Rhizopus stolonifer*, *Rhizoctonia solani*, and *Alternaria alternata* [25,44] and explains why both oils produced strong inhibition of mycelial extension yet, in most cases, failed to prevent regrowth after reinoculation: sublethal exposure appears to alter membrane permeability enough to arrest growth without triggering irreversible cell death, consistent with a fungistatic rather than fungicidal effect [45]. This transient efficacy is consistent with the high volatility of essential oils, which promotes rapid evaporation and degradation via oxidation, isomerization, and polymerization, reducing the concentration of active compounds over time [46]—a pattern that parallels reports of peppermint EO’s inhibitory effect against *F. oxysporum* weakening between days three and twelve as the oil degrades [47] and mirrors the decline we observed in inhibition of *F. avenaceum* (100% to 95%) and *A. alternata* (100% to 73%) at the highest PEO concentration between early and late assessment days.

Clove PE behaved differently. Unlike the other treatments, CPE was fungicidal against both isolates, an effect we attribute to its high eugenol content (52.88%), which is reported to cause more extensive and irreversible membrane disruption than thymol or menthone at sufficient concentration, driving cell collapse rather than transient permeabilization. Rosemary extract, dominated by eucalyptol and camphor rather than phenolics, produced comparatively weaker and slower-developing inhibition, particularly against *A. alternata*, consistent with a milder, non-phenolic mode of membrane interaction. The greater resistance of *A. alternata* relative to *F. avenaceum* across all essential oil and extract treatments may reflect structural rather than purely chemical differences: the melanized, more heavily pigmented hyphae characteristic of *Alternaria* spp. are thought to confer additional protection against oxidative and membrane stress, whereas *Fusarium* spp. hyphae may allow faster penetration of lipophilic terpenes. The transient stimulation of *A. alternata* growth observed after reinoculation at the lowest EO concentration is consistent with hormesis, a low-dose stimulatory response reported previously for other necrotrophic pathogens exposed to subinhibitory concentrations of antifungal compounds rather than an artefact of the assay [25,44,45,46,47,48].

The commercial biological products relied on different mechanisms entirely, which explains their distinct reinoculation behavior. Mycostop, based on *Streptomyces griseoviridis*, was the most effective commercial treatment and, notably, was the only commercial product besides CPE to retain substantial inhibitory activity after reinoculation, reaching 84% and 95% inhibition of *F. avenaceum* and *A. alternata*, respectively, at 7 DAI. This is consistent with reports that *S. griseoviridis* inhibits fungi not only via secreted antibiotics and hydrolytic enzymes [49] but also by forming direct physical contact with fungal mycelium, reducing host cell turgor and potentially being carried over with transferred mycelial fragments to continue colonizing and suppressing growth on fresh medium [50]—a mechanism broadly consistent with the antagonistic activity reported for other *Streptomyces* strains against diverse fungal pathogens [34]. *Aegis* and *Asir Fruit*, which rely on arbuscular mycorrhizal fungi (*Rhizoglomus irregularis*, *Funneliformis mosseae*) and, for Asir Fruit, *Trichoderma koningii* and *Bacillus megaterium*, showed weaker and slower-developing inhibition (68% and 63% against *F. avenaceum*, and 73% and 46% against *A. alternata*, at 7 DAI) that did not persist after reinoculation. However, in vitro assays are inherently unsuitable for evaluating AMF-based products, as their biocontrol mechanisms rely on establishing mutualistic symbiosis with host plants. Therefore, the weaker direct in vitro inhibition observed here must not be interpreted as evidence of poor field efficacy; the products’ performance could be expected to be better [51]. These organisms act primarily indirectly, through competition for space and nutrients and induction of host plant defense responses—mechanisms that depend on an established symbiotic or rhizosphere interaction and are unlikely to manifest, or to transfer along with a mycelial plug, in a direct antibiosis assay of this kind. This distinction matters practically: our in vitro screen is best suited to detecting direct antibiosis and likely underestimates the field performance of AMF-based products, whose benefits typically depend on root colonization and induced resistance rather than direct pathogen contact [50].

Taken together, these results indicate that the treatments fall into two functionally distinct groups: fast-acting, membrane-disrupting agents (the essential oils and plant extracts), whose efficacy is concentration-dependent and, except for clove extract, reversible, and a slower-acting biological agent (Mycostop) whose activity depends on sustained microbial colonization rather than a single chemical mode of action. This distinction has practical implications for deployment: essential oils and clove extract may suit rapid, direct-contact applications such as post-harvest dips, whereas Streptomyces-based products may be more appropriate where sustained protection through tissue colonization is needed. Because the isolates used here originated from Lithuania, and because the composition of essential oils and plant extracts is known to vary with growing conditions, harvest stage, and extraction method [22], these mechanistic interpretations should be tested against isolates from other regions and against oils of differing chemotypes before their field applicability can be confirmed. The findings of this study demonstrate that the tested essential oils, plant extracts and commercial products exhibit in vitro antifungal activity. Therefore, future studies should assess the efficacy of these natural products under different environmental conditions and against a broader range of plant pathogens. Expanding the evaluation to additional essential oils, plant extracts, and microbial products may facilitate the identification of promising alternatives for integrated and sustainable plant disease management. Although the fungal isolates used in this study originated from Lithuania, they represent economically important plant pathogens with broad geographic distribution.

## 4. Materials and Methods

### 4.1. Pathogenic Fungal Isolates

The single-spore isolates were obtained from the isolate collection of the Lithuanian Research Centre for Agriculture and Forestry (LAMMC), Institute of Horticulture, Laboratory of Plant Protection. Both isolates were identified previously: *Fusarium avenaceum* (LT-Fus-5, host: strawberry fruit) [52] and *Alternaria alternata* (LT-Alt-2, host: strawberry leaf) [53,54]. The isolates were maintained on potato dextrose agar (PDA, Liofilchem, Roseto degli Abruzzi, Italy) at 4 °C, subcultured onto fresh PDA plates 7 days prior to the experiment, and then incubated at 22 °C in the dark.

### 4.2. Essential Oil Hydrodistillation

Peppermint (*Mentha × piperita* L.) and thyme (*Thymus vulgaris* L.) essential oils (EOs) were extracted by hydrodistillation using a Clevenger distillation system (Glassco, Ambala Cantt, India). A total of 100 g of dried peppermint or thyme leaves (grown in LAMMC) were placed in a 2-liter round-bottomed flask, and 600 mL of distilled water was added. The peppermint leaves were dried at 40 °C for 2 days. The flask was placed on a heating mantle (Labbox LBX HM01, Barcelona, Spain) and brought to a boil. The condensate was collected. The duration of the hydrodistillation process was 2 h at atmospheric pressure [55]. The volatile constituents of peppermint and thyme EOs were analyzed using gas chromatography–mass spectrometry. The analysis was performed on a GC-2010Plus/GCMS-QP2010 Ultra system (Shimadzu, Kyoto, Japan) equipped with an Rxi-5MS capillary column (30 m × 0.25 mm, 0.25 μm film thickness) (Restek, Bellefonte, PA, USA). The carrier gas flow rate was 1 mL min^−1^, and the injector temperature was 250 °C. The column temperature was programmed to increase from 50 °C to 160 °C at 5 °C min^−1^, followed by a ramp to 250 °C at 10 °C min^−1^. Samples were introduced in split mode at a 1:20 ratio. Mass spectra were recorded in electron impact mode at 70 eV and 220 °C [24,56]. Results of volatile compounds of essential oils have been provided in Table 3 and reported in our previous studies [24,41]. Essential oils were kept at −20 °C until the experiments.

### 4.3. Subcritical CO_2_ Extraction

Rosemary (*Rosmarinus officinalis* L.) and clove (*Syzygium aromaticum* L.) plant extracts were obtained through subcritical CO_2_ extraction. An amount of 5 kg of dried rosemary leaves and clove buds were placed in the extraction vessel, and the extraction was performed for 6 h at 42 bar and 10 °C. The extracts were collected and stored at 4 °C until further analysis [57]. The volatile constituents of the extracts were subsequently characterized by gas chromatography–mass spectrometry (GC–MS), as mentioned earlier. Results of volatile compounds of plant extracts are provided in Table 3 and reported in our previous studies [4,56]. Plant extracts were kept at +4 °C until the experiments.

### 4.4. Antifungal Activity of Alternative Treatments

The antifungal activity of thyme and peppermint essential oils, as well as clove and rosemary plant extracts, was tested against *A. alternata* and *F. avenaceum* pathogens using the agar incorporation method assay. In addition, commercial biological products were used as a comparison. The experiments were conducted in vitro. The antifungal treatments are provided in Table 4. The concentrations of the commercial products were selected based on the manufacturer’s recommendations for use in horticultural crops. Concentrations were selected based on preliminary inhibition tests in previous studies. Detailed information is provided in Table 4.

The experiment was carried out in four replicates (completely randomized design). After autoclaving and cooling the PDA to 40 °C, the tested concentrations (Table 4) were added to the media supplemented with 0.1% (*v*/*v*) Tween 20 (Sigma Aldrich, Steinheim, Germany), well mixed, and poured into the Petri dish. Mycelial plugs (6 mm in diameter) of 7-day-old cultures of *Fusarium avenaceum* and *Alternaria alternata* isolates were placed at the center of the Petri dish, with the mycelium downward. Control plates were prepared identically, except that no additives were used. The Petri dish was incubated at 22 °C in the dark.

Mycelial growth was measured at 2, 4, and 7 days after inoculation (DAI). The average mycelial diameter (mm) was measured at two perpendicular axes in the Petri dish, and inhibition was calculated using the following formula: inhibition (%) = (average colony diameter of the pathogen in the control (mm) − average colony diameter of the pathogen in the treatment (mm))/average colony diameter of the pathogen in the control (mm) × 100% [4,58].

### 4.5. Reinoculation

Reinoculation was performed to evaluate the recovery (to test if they are viable) of the fungi after treatment exposure at the end of the antifungal activity experiment. Reinoculation was used to evaluate whether there was antifungal activity of the tested treatments. The observations after the reinoculation were as follows: fungistatic (inhibited the growth of fungi without killing them, the fungi started to grow) or fungicidal (directly killing fungal cells; total absence of growth).

At 7 DAI, mycelial fragments from each treatment were transferred into new PDA without any additives. From each treatment, 6 mm diameter plugs of mycelium were cut from the Petri margin of the inhibited colony and transferred to a new Petri dish. For treatments showing complete inhibition, the original mycelial plug, which was in the center of the Petri dish, was taken precisely, without transferring previous media with the treatment. The plugs were placed mycelium side down onto new PDA (four replicates, completely randomized design). The plates were incubated at 22 °C, and mycelial growth was measured 2 DAI. During each evaluation, colony diameter was determined by measuring two perpendicular axes [4].

### 4.6. Processing of Data

All data obtained in the study were analyzed using one-way ANOVA in SAS v7.1 Enterprise Guide (SAS Institute Inc., Cary, NC, USA). The results are presented as mean values ± standard error. Differences between treatments were evaluated using Duncan’s multiple range test at a significance level of 0.05. The standard error of the mean was calculated using Microsoft Excel [55].

## 5. Conclusions

This study contributes to the literature by providing a standardized, side-by-side comparison of essential oils, plant extracts, and commercial biological products against *Fusarium avenaceum* and *Alternaria alternata* under identical in vitro conditions. Clove plant extract was uniquely fungicidal, while thyme essential oil and Mycostop were the most effective treatments in their respective categories, with Mycostop showing sustained fungistatic and carry-over antagonistic activity. These findings support further evaluation of clove extract and thyme oil as candidates for integrated disease management, alongside continued development of *Streptomyces*-based biocontrol, under greenhouse and field conditions and against a wider range of fungal pathogens and isolate origins.

## Figures and Tables

**Table 1:** In vitro inhibition of mycelial growth (%) of *Fusarium avenaceum* and *Alternaria alternata* 7 DAI at the highest tested concentrations of the treatments.

Treatment	Concentration	*F. avenaceum*	*A. alternata*
Peppermint EO	2000 μL/L	95.63 b	72.95 b
Thyme EO	1000 μL/L	100 a	100 a
Clove PE	2000 μL/L	100 a	100 a
Rosemary PE	2000 μL/L	57.34 f	21.26 d
Aegis	1 tablet/L	67.76 d	73.39 b
Mycostop	0.1 g/L	83.46 c	94.68 a
Asir Fruit	1 tablet/L	62.73 e	45.57 c

Different letters within each fungal species indicate statistically significant differences among treatments, as determined by one-way ANOVA followed by Duncan’s test (*p* < 0.05).

**Table 2:** In vitro inhibition of mycelial growth (%) of *Fusarium avenaceum* and *Alternaria alternata* relative to untreated control.

Treatment	Concentration	*F. avenaceum*			*A. alternata*		
		2 DAI	4 DAI	7 DAI	2 DAI	4 DAI	7 DAI
Control	-	0 c	0 d	0 d	0 b	0 b	0 b
Peppermint EO	1200 μL/L	73.02 b	45.60 c	36.15 c	100 a	94.92 a	62.80 a
Peppermint EO	1600 μL/L	100 a	86.71 b	72.77 b	100 a	95.20 a	60.39 a
Peppermint EO	2000 μL/L	100 a	100 a	95.63 a	100 a	100 a	72.95 a
Control	-	0 b	0 c	0 c	0 c	0 b	0 b
Thyme EO	200 μL/L	93.55 a	66.71 b	43.15 b	52.73 b	−2.86 b	−29.94 c
Thyme EO	600 μL/L	100 a	99.29 a	89.72 a	100 a	96.79 a	86.89 a
Thyme EO	1000 μL/L	100 a	100 a	100 a	100 a	100 a	100 a
Control	-	0 b	0 b	0 b	0 b	0 b	0 b
Clove PE	1200 μL/L	100 a	100 a	100 a	100 a	100 a	100 a
Clove PE	1600 μL/L	100 a	100 a	100 a	100 a	100 a	100 a
Clove PE	2000 μL/L	100 a	100 a	100 a	100 a	100 a	100 a
Control	-	0 c	0 c	0 b	0 c	0 c	0 b
Rosemary PE	1200 μL/L	58.60 b	58.17 ab	57.25 a	35.17 b	14.41 b	3.06 b
Rosemary PE	1600 μL/L	60.47 ab	55.48 b	56.78 a	70.34 a	31.36 a	14.65 a
Rosemary PE	2000 μL/L	64.19 a	60.32 a	57.34 a	72.41 a	40.11 a	21.26 a
Control	-	0 c	0 c	0 d	0 c	0 d	0 d
Aegis	1 tablet/L	45.35 a	38.85 b	67.76 b	15.38 b	34.94 b	73.39 b
Mycostop	0.1 g/L	14.60 b	63.06 a	83.46 a	86.01 a	87.68 a	94.68 a
Asir Fruit	1 tablet/L	12.56 b	41.47 b	62.73 c	−2.76 c	12.15 c	45.57 c

For each treatment, differences within each column were evaluated using one-way ANOVA followed by Duncan’s test (*p* < 0.05). Different letters indicate significant differences. Negative inhibition values indicate mycelial growth stimulation relative to the control, reflecting a low-dose stimulation phenomenon (hormesis).

**Table 3:** The essential oils’ and extracts’ chemical composition.

Name	Plant Source	Country	Major Component and Concentration (%)	Reference
Peppermint essential oil	*Mentha × piperita* L.	Lithuania	Menthone (44.56%), isomenthone (12.81%), pulegone (10.74%), menthol (7.95%)	[24]
Thyme essential oil	*Thymus vulgaris* L.	Lithuania	thymol (52.22%), p-cymene (12.37%), γ-terpinene (8.39%), linalool (4.26%)	[41]
Rosemary extract	*Rosmarinus officinalis* L.	Lithuania	Eucalyptol (41.28%), camphor (16.62%), α-pinene (8.92%)	[4]
Clove extract	*Syzygium aromaticum* L.	Lithuania	Eugenol (52.88%), eugenol acetate (21.95%), *trans*-caryophyllene (17.80%)	[56]

**Table 4:** Characteristics of the tested treatments used in the in vitro experiments.

Treatment	Commercial Name	Main Ingredients	Coding	Concentration
Essential oils	-	*Mentha × piperita* L.: Menthone, isomenthone, pulegone, menthol, eucalyptol [24]	PEO1200	1200 μL/L
			PEO1600	1600 μL/L
			PEO2000	2000 μL/L
	-	*Thymus vulgaris* L.: thymol, p-cymene, γ-terpinene, linalool, carvacrol [41]	TEO200	200 μL/L
			TEO600	600 μL/L
			TEO1000	1000 μL/L
Plant extracts	-	*Syzygium aromaticum* L.: eugenol, eugenol acetate, *trans*-caryophyllene [4]	CPE1200	1200 μL/L
			CPE1600	1600 μL/L
			CPE2000	2000 μL/L
	-	*Rosmarinus officinalis* L.: eucalyptol, camphor, α-pinene, α-terpineol [56]	RPE1200	1200 μL/L
			RPE1600	1600 μL/L
			RPE2000	2000 μL/L
Biological products ***	Aegis tablet *	*Rhizoglomus irregularis* BEG72, 500 sp/tablet (100 spores/g)	Aegis	1 tablet/L
	Mycostop **	*Streptomyces griseoviridis* K61, 5 × 10^8^ CFU/g	Mycostop	0.1 g/L
	Asir Fruit *	*Rhizoglomus irregularis* BEG72 50 spores/g *Funneliformis mosseae* BEG234 50 spores/g *Trichoderma koningii* TK7, 1 × 10^7^ CFU/g *Bacillus megaterium* MHBM77, 1 × 10^7^ CFU/g *Bacillus megaterium* MHBM06, 1 × 10^7^ CFU/g NPK 8-6-4	Asir Fruit	1 tablet/L

CFU: Colony-forming unit, * Agrotecnologias Naturales S.L. (ATENS), Spain. ** Danstar Ferment AG, Switzerland. *** commercial products obtained from manufacturers.

## Data Availability

The data presented in this study are available upon request from the corresponding author.
